# Hypoplastic coronary arteries in a child with a mutation in *Notch1*: A case report

**DOI:** 10.1097/MD.0000000000021355

**Published:** 2020-08-14

**Authors:** Xiaoqing Shi, Jianxin Liu, Jinlin Wu, Yimin Hua, Kaiyu Zhou, Yifei Li

**Affiliations:** a Key Laboratory of Birth Defects and Related Diseases of Women and Children of MOE, Department of Pediatrics, West China Second University Hospital, Sichuan University, Chengdu, Sichuan, China; b State Key Laboratory of Oral Diseases, National Clinical Research Center for Oral Diseases, West China Hospital of Stomatology, Sichuan University, Chengdu, Sichuan, China.

**Keywords:** genomic sequence, hypoplastic coronary artery disease, *NOTCH1* gene

## Abstract

**Rationale:**

Coronary artery abnormalities are usually of major significance in clinical cardiology and cardiac surgery departments due to associated myocardial ischemia, myocardial infarction, and sudden cardiac death. Among them, anatomical malformations account for most coronary artery abnormalities. However, hypoplasia of the coronary artery is a rare type of coronary artery without any genetic screening information.

**Patient concerns:**

A 10-year-old boy suffered severe chest pain, and a subsequent syncope occurred.

**Diagnosis and intervention:**

The boy complained of significant chest pain with syncope. Computerized tomography (CT) angiography scanning showed that the left coronary artery was dominated by abnormal origins and dramatically narrow artery lesions. Moreover, cardiac magnetic resonance imaging (MRI) confirmed myocardial ischemia. Cardiac catheterization confirmed that this was an extremely rare hypoplastic coronary case. Finally, a mutation was identified in *NOTCH1* c.1023C>A for the first time.

**Outcomes:**

The boy was discharged after completing all examinations and was forbidden to play any kind of sport activity while waiting for heart transplantation.

**Lessons:**

Hypoplastic coronary artery diseases have only been reported within very limited cases. This is the only report that has identified hypoplasia in 3 epicardial major coronary arteries. In addition, this is the first case to provide evidence between *NOTCH1* genetic disorder and hypoplastic coronary artery disease in the clinic.

## 1 Introduction

Hypoplastic coronary artery disease (HCAD) is an extremely rare congenital coronary artery malformation with underdeveloped major epicardial artery branches.^[1]^ As in previous reports, 1% of the whole population has coronary artery abnormalities, while only 2.2% of patients with coronary artery abnormalities have been identified on postmortem analysis. However, significantly limited cases have been reported in living people with HCAD.^[2]^ Only 1 article provided a long-term follow-up.^[3]^ Unfortunately, the outcomes of HCAD patients are poor, and sudden cardiac death occurs in all cases.^[2]^ In addition, no research has demonstrated any clues on the genetic mechanism of HCAD. Here, we report the youngest patient diagnosed with HCAD. Moreover, a mutant site of *NOTCH1*, c.1023C>A, was identified among all suspected sites after whole-genome sequencing. The patient and his parents provided informed consent for publication of the case.

## 2 Case presentation

A 10-year-old boy presented to the emergency department after losing consciousness while running at school 2 days ago. He experienced dyspnea and palpitations before syncope. After recovery of consciousness, the boy complained of severe chest pain which disappeared in half an hour. Witness's descriptions suggested no tonic movement, such as seizure activity. The boy and his parents definitely confirmed that he had never experienced syncope before. The patient denied any alcohol or drug ingestion. There was no related family history of any heart diseases, including cardiomyopathy, acute coronary artery syndrome, and sudden cardiac disease.

When he arrived at the hospital, there was no significant clinical manifestation. The physical examination was unremarkable. Laboratory studies demonstrated a normal range of C-reactive protein (CRP), erythrocyte sedimentation rate (ESR), and procalcitonin (PCT) as well as electrolytes, glucose, and hemoglobin. Surprisingly, no evidence demonstrated myocardial injuries with normal cTnI and brain natriuretic peptide (BNP). Additionally, autoantibodies were negative in this patient.

According to the diagnostic procedure, electrocardiogram (ECG) showed depression of the ST-T segment. Echocardiography failed to identify any abnormalities in heart structure or cardiac function. Then, computerized tomography (CT) angiography revealed a left dominated coronary artery with abnormal origin of the right coronary artery. In addition, it also reported a dramatically narrow artery lesion among global coronary arteries. Moreover, myocardial ischemia was confirmed by cardiac magnetic resonance imaging (MRI).

**Figure 1:**
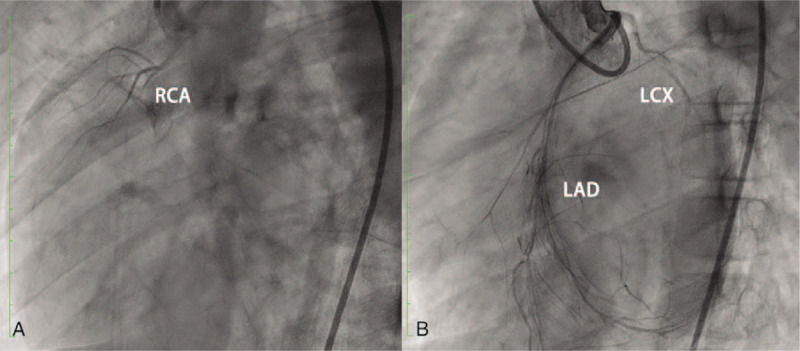
Catheter angiography demonstrated hypoplasia of 3 major epicardial coronary arteries. All the 2 images had been taken in a position of LAO 60° and CRA 20°. An image identified the shortness and narrow without sufficient distribution of RCA. B image showed the hypoplasia of LAD and LCX. The LCX was similar as RCA, with narrow lumen and limited distributions. While LAD processed enough length but severely narrow lumen found with stiff vessel structure. CRA = cranial right anterior view, LAD = left anterior descending artery, LAO = left anterior oblique view, LCX = left circumflex, RCA = right coronary artery.

After 5 days, the patient was admitted to the cardiac department. Cardiac catheterization was performed. Significantly, abnormal coronary artery formation was identified. All 3 major epicardial coronary artery branches revealed narrowed, thinning, and stiff structures. The right coronary artery (RCA) originated from the trunk of the aorta, with a diameter smaller than 1 mm and a length <20 mm, indicating an undeveloped coronary vessel (Fig. 1A). During left coronary artery angiography, the origin of the left anterior descending (LAD) artery was normal with a 3.5 mm diameter, but this lumen was only 8 mm and became narrow with complex small vessels on the surface of the heart. The left circumflex (LCX) branch failed to display well during angiography (Fig. 1B). Therefore, according to angiography, all 3 major epicardial coronary artery branches were identified as hypoplastic vessels.

Previous cases failed to provide any genetic and molecular clues for HCAD. However, some basic research has focused on coronary artery development. Several genetic factors have been reported that might be related to HCAD. Finally, due to genomic sequencing, a new mutation site was identified in *NOTCH1* c.1023C>A, with a score of 0.965 in evaluating loss of function of the gene using Mutation Taster (disease causing, http://www.mutationtaster.org), and 0.811 in evaluating protein function revealing possible damage using PolyPhen-2 (http://genetics.bwh.harvard.edu/pph2).

## 3 Discussion

HCAD was first reported in the 1970s and was identified as one or more major branches of the coronary artery presenting a severely narrow lumen.^[4]^ To date, fewer than 30 cases have been described in the literature. Initially, HCAD could only be diagnosed by necropsy after sudden cardiac death occurred in such patients.^[3,5,6]^ Recently, a few patients had undergone catheter angiography, and live images for HCAD were obtained. In summary, the median age at diagnosis of HCAD is 30.5 years (IQR 26–30.5).^[1,2]^ Syncope and sudden cardiac death are usually described as the first experienced clinical symptoms. Based on current reports, 16 of 26 cases had RCA abnormalities, while 15 and 9 cases reported LCX and LAD hypoplasia, respectively. This patient suffered syncope at only 10 years old, which is a very early-onset case. Referring to his angiography results, 3 major epicardial coronary artery branches demonstrated hypoplasia, resulting in the phenotype of this patient.

The outcomes of all previously reported HCAD cases were unfortunately poor, and no patients survived. Available therapies for HCAD are limited. Bypass surgery would not be a wise choice because the diffuse distribution of coronary vessels was abnormal. For this issue, heart transplantation is the only and most effective treatment alternative. Recently, implantable cardioverter-defibrillator (ICD) was referred to HCAD patients due to the high risk of threatened ventricular arrhythmia.^[1]^ However, the long-term results were absent, and it would still take time to evaluate the efficacy of ICD in HCAD.^[5]^

With the rapid development of genetic technology, the price of genomic analysis has dropped to approximately 100 USD, which has helped physicians obtain a better understanding of the genetic and molecular mechanisms of a large number of diseases. However, previous reports were published a decade ago, so genetic information was absent. This case is one of the youngest patients who received HCAD diagnosis, with hypoplasia of 3 major epicardial coronary artery branches. Therefore, genetic mutations were suspected. A series of basic studies on coronary artery embryogenesis and genes related to smooth muscle progenitor cells and endothelial cells were identified,^[7]^ including *NOTCH1*, *NFATc1*, *TBX1*, *VEGF*-a, *VEGF*-c, *FGF* family, *SHH*, *CXCL12*, *EFNA1*, *EFNA2*, *SEMA* family, *WT1*, *NR2F2*, *TCF21*, *TBX18*, *GATA4*, *RXRa*, *IGF2*, etc.^[1]^ According to our genomic analysis, a mutation site located in *NOTCH1* c.1023C>A was found. NOTCH signaling was demonstrated to be involved in promoting coronary artery endothelium fate, and del Monte et al^[8]^ confirmed that *NOTCH1* participated in the development of the coronary artery. Based on Mutation Taster, this mutation is a disease-causing site. Therefore, this is the first pathogenic-like mutation reported among HCAD cases.

In conclusion, this case is one of the youngest patients who received an HCAD diagnosis. In addition, the patient was treated as the most severe case with 3 major abnormal branches. Genetic analysis provides a suspected mutation site as *NOTCH1* c.1023C>A.

## Author contributions

**Conceptualization:** Xiaoqing Shi, Yimin Hua, Kaiyu Zhou, Yifei Li.

**Data curation:** Xiaoqing Shi, Yimin Hua.

**Formal analysis:** Xiaoqing Shi, Jianxin Liu.

**Investigation:** Jinlin Wu, Yimin Hua, Yifei Li, Jianxin Liu.

**Methodology:** Yimin Hua, Yifei Li.

**Project administration:** Jinlin Wu, Yifei Li.

**Supervision:** Yifei Li.

**Validation:** Kaiyu Zhou, Jianxin Liu.

**Visualization:** Kaiyu Zhou.

**Writing – original draft:** Xiaoqing Shi, Jinlin Wu, Kaiyu Zhou.

**Writing – review & editing:** Kaiyu Zhou, Yifei Li.

## Correction

When originally published, the first author's name, Xiaoqing Shi, appeared incorrectly as Xiaoqing XS. The abbreviation of that name in the footnote also appeared incorrectly as XA instead of XS. Both of these have been corrected.
